# Development of a quick and simple detection methodology for foot-and-mouth disease virus serotypes O, A and Asia 1 using a generic RapidAssay Device

**DOI:** 10.1186/1743-422X-10-125

**Published:** 2013-04-22

**Authors:** Ming Yang, Melissa Goolia, Wanhong Xu, Hilary Bittner, Alfonso Clavijo

**Affiliations:** 1 National Centre for Foreign Animal Disease, 1015 Arlington Street, Winnipeg, Manitoba, R3E 3M4, Canada; 2 Curent address: Pan American Health Organization (PAHO/WHO), Av. Presidente Kennedy, 7778, Duque de Caxias, Rio de Janeiro, CEP 25040-004, Brazil

**Keywords:** Foot-and-mouth disease virus, Rapid viral antigen detection, Lateral flow immunochromatographic strip test

## Abstract

**Background:**

Outbreaks of Foot-and-mouth disease (FMD) have resulted in tremendous economic losses. Thus, the development of a rapid and easily performed test for FMD detection is important for controlling a FMD outbreak and containing its spread. The purpose of this project is to develop a lateral flow immunochromatographic (LFI) strip test for rapid detection of FMD virus serotypes O, A and Asia 1.

**Methods:**

Specific monoclonal antibodies (mAbs) against each serotype were produced and used as the capture mAbs. A serotype independent mAb was selected and used as the detection mAb with the aim of subsequently developing a multi-serotype strip test. A new generation of the generic RapidAssay Device (gRAD) was used for the test.

**Result:**

Each strip test can specifically detect the FMDV O, A or Asia 1 viruses, but not other vesicular disease viruses. The LFI strip tests for serotypes A and Asia 1 were able to identify all tested serotype A (n= 39) and Asia 1 field isolates (n=17). Whereas the test for serotype O detected 45 out of 46 field isolates. The sensitivity of this strip test was comparable with the double antibody sandwich ELISA for viral antigen detection. All vesicular fluid and epithelium samples collected from experimentally infected animals with serotype O, A and Asia 1 were identified as positive by the LFI strip test. Swab samples (n=11) collected over the lesion area from experimentally inoculated animals (serotype A) were examined. All of them demonstrated positive results using the LFI serotype A strip test and double antibody sandwich (DAS) ELISA.

**Conclusions:**

The ability of strip tests to produce rapid results and high specificity makes it a valuable tool for early detection of FMDV O, A and Asia 1 in the field.

## Introduction

Foot-and-mouth disease (FMD) remains one of the world’s most widespread epizootic and highly contagious animal diseases. More than 100 countries are not yet recognized as officially free of FMD by the World Organisation for Animal Health (OIE). The rapid spread of the disease in affected animals generates significant economic losses worldwide. Based on serological tests, FMD virus (FMDV) is recognized as seven serotypes: O, A, C, Asia 1, SAT 1, SAT 2 and SAT 3. There are a large number of subtypes within each serotype due to extensive genetic and antigenic variation among them [1,2]. Among the seven serotypes of FMDV, O and A are the most widespread and currently found in Africa, the Middle East, Asia, limited area of South America and sporadically in Europe. Asia 1 is primarily found in Asia, periodically into the Middle East and occasionally Europe [3]. SAT 1, 2, and 3 are primarily restricted to Africa. Outbreaks of SAT 1 and 2 in the Middle East have been reported [4,5]. Viruses of serotype C now appear extremely rare or may even have totally disappeared; the last confirmed case was the Amazon region of Brazil in 2004 and Kenya in 2005 [6,7]. The occurrence of FMD outbreak indicates the need to develop rapid tests for early diagnosis in affected areas. The rapid virus identification has important clinical, economic, and epidemiological implications.

Various laboratory methods are currently available for FMDV detection, including virus isolation, real-time reverse-transcription (RRT) PCR and double antibody sandwich (DAS) enzyme-linked immunosorbent assay (ELISA). Although the ELISA is relatively simple and easy to perform, it is difficult to perform the test in the field and take hours to obtain results. These assays require laboratory operations, well-trained personnel, and special equipment/facilities. It would be impractical and excessively costly for all countries to maintain a diagnostic laboratory with full capabilities for confirmatory diagnosis of FMD. The lateral flow immunochromatographic (LFI) strip tests have been widely used for the diagnosis of many contagious diseases and the detection of bioactive molecules, such as hormones, haptens, and many others [7-9]. The LFI strip test has many advantages including low cost, short timeline for development, ease of performing and result interpretation, minimum amount of training for personnel and no special equipment required. The test can be performed rapidly on-site during a major epidemic. Recently, LFI strip tests have been efficiently applied to the detection of specific antibodies against FMDV non-structural protein [10] and FMDV serotype O [11]. The LFI strip tests have also been developed for the detection of non-serotype specific FMDV [12,13]. The availability of the non-serotype specific strip test would allow for the on-site diagnosis of suspected FMD outbreaks. A limiting factor for this non-serotype specific strip test is that they are unable to identify the serotype of FMDV, thus reducing their potential benefit in endemic countries, where rapid identification of the serotype may be essential to disease control [14]. The development of the LFI strip test for single serotypes will be useful for rapid detection in a secondary outbreak situation in which the serotype was identified from the initial outbreak. It can also be used to provide evidence of the disease spreading around the initial outbreak, thus reducing the delay in specimen submission for testing. The LFI strip tests were developed for rapid testing of FMDV serotypes A and Asia 1 [15,16]. A strip test to identify FMDV serotypes O, A, and Asia 1 has been reported [15]. However, polyclonal sera from rabbits and guinea pigs were used as the capture and detection antibodies in their study. The use of mAbs in the LFI test increases the specificity, accuracy, consistency, efficiency of diagnosis and is easy to standardize compared with polyclonal antibodies.

Of the seven serotypes, serotypes A, O and Asia are more prevalent. The aim of this study was to produce specific mAbs against FMDV O, A and Asia 1 and develop serotype-specific LFI strip tests for the detection of FMDV serotypes O, A and Asia 1 using a new generation of the generic RapidAssay Device (gRAD). In a general LFI strip test, the specific detection antibody is immobilized on the surface of the membrane so each test device is custom made. This requires every single test to be normalized, standardized, and validated. The advantages of using the gRAD are that (1) there is no need to spray specific antibodies or reagents to the membrane, (2) it is ready to use for different tests with selected specific antibodies, (3) the biotin-streptavidin system will possibly enhance and improve the sensitivity of the traditional LFI and (4) it is commercially available, which makes assay development and standardization easier. In addition, the pre-made gRAD is stable in storage for 2 years at room temperature. In this study, a serotype independent mAb produced previously [17] was used as the detection mAb to establish a foundation for future development of a multiple serotype test in a single device. The performance of each of the newly developed LFI strip tests for FMD serotypes O, A, and Asia 1 was compared with other methodologies for viral antigen detection. Due to its ability to produce rapid results, the availability of the strip test would be useful for early diagnosis of FMDV O, A and Asia 1 in an endemic situation or during an epidemic. The LFI strip test can be potentially considered as a primary key diagnostic test on-site.

## Results

### Monoclonal antibody production

The fusions were performed using mouse spleen cells inoculated with BEI-inactivated viruses O1/Campos, A_22_ /Iraq and Asia 1/Shamir. Three fusions for each serotype were performed and allowed for the production of 14 hybridomas for FMDV serotype O, 15 hybridomas for serotype A and 2 hybridomas for serotype Asia 1 respectively. After the subcloning, the mAbs were designated and their isotypes were characterized. The mAbs were examined for their reactivity and specificity against different FMDV serotypes using FMDV serotype specific DAS ELISAs. The results indicate that all mAbs are FMDV specific without cross reactivity against other vesicular disease viruses (data not shown).

### Selection of detection mAb

**Figure 1:**
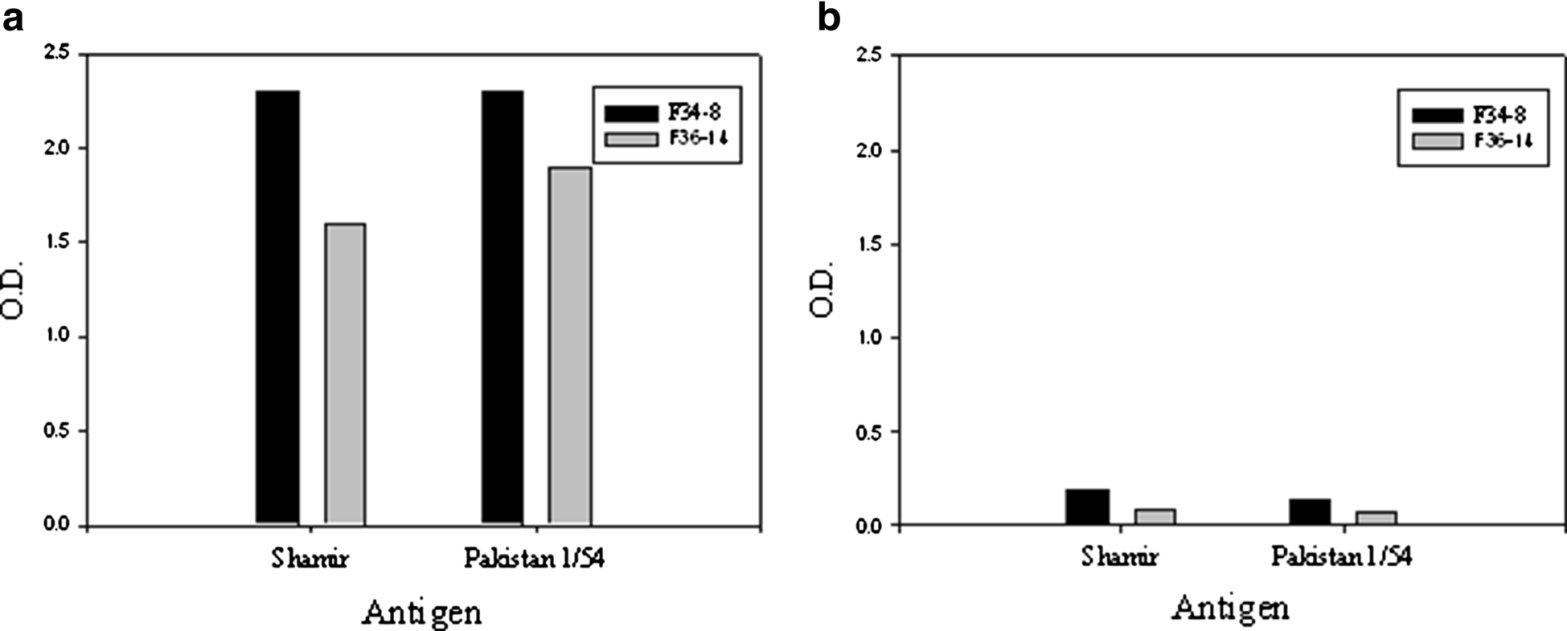
**Selection of detection mAbs using a DAS ELISA.** Two purified mAbs (F34Asia-8 and F36Asia-14) were coated onto microtiter plates. Then the FMDV Asia 1/Shamir and Asia 1/Pakistan 1/54 antigens were added to the plates. The antigen binding was detected with biotinylated serotype independent mAbs: (**a**) mAb F21-140SO and (**b**) mAb F14-12SA. The binding was detected with an avidin-HRP conjugate (1:2000).

Since the final goal is to develop a single strip test for FMDV multi-serotype antigen detection, two serotype independent FMDV specific mAbs, F14-12SA and F21-140SO [17] were examined using a DAS ELISA to determine their suitability as a detection antibody. FMDV serotype Asia 1 virus was selected as a representation. Two purified Asia 1 specific mAbs were coated onto microtiter plates as the capture antibody. The antigen binding was detected using two biotinylated serotype independent mAbs as the detection mAb. The ELISA results demonstrated excellent reactivity with mAb F21-140SO (Figure 1a), while the other serotype independent mAb F14-12SA was unable to detect the Asia 1 antigen in the DAS ELISA (Figure 1b). Thus, the mAb F21-140SO was selected and used as the detection mAb in the strip test. This mAb was purified and conjugated to colloidal gold particles using Naked Gold in a Box™ .

### Selection of capture mAbs

DAS ELISAs were used to select capture mAbs matching the selected detection mAb for strip test development. The ability of 15 serotype A specific mAbs was examined to detect the different serotype A field isolates. A total of 39 FMDV serotype A field isolates were tested. The results indicated that the mAb F66A_22_-14 reacted with all of the isolates. Therefore the mAb F66A_22_-14 was selected and used in the serotype A LFI strip test.

Two Asia 1 specific mAbs F34Asia-8 and F36Asia-14 were tested using a DAS ELISA. Monoclonal Ab F34Asia-8 demonstrated low binding affinity to Asia 1/IRN31/2004 and failed to react with Asia 1/KRG1/2004 (data not shown). While the mAb F36-14 failed to react with an Asia 1/IND 8/79 isolate and showed lower binding affinity to five newly isolated FMDV Asia 1 strains. The use of single mAb may not be able to recognize all isolates when antigenic evolution occurs. To overcome the limitation of a single mAb binding one epitope and increase the detection strain variety, it was decided to combine two Asia 1 specific mAbs as the capture mAbs. The mAb F34Asia-8 showed neutralization activity against Asia 1/Shamir, while the mAb F36Asia-14 did not, suggesting that the binding sites of these two Asia 1 specific mAbs were different from each other.

For serotype O capture mAb selection, the reactivity of 14 serotype O specific mAbs with 46 serotype O field isolates were examined using a DAS ELISA. One (F1O-2-5) out of 14 serotype O specific mAbs reacted with 45 out of 46 serotype O isolates, but failed to bind with O/ECU4/10. It is possible that mutations occurred for O/ECU4/10 on mAb (F1O-2-5) binding site. Since the mAb F1O-2-5 is the one that reacted with most of serotype O isolates, it was selected and used as the capture mAb in the LFI strip test. All mAbs selected for the LFI strip test failed to react with the denatured viruses using an indirect ELISA, which suggests that the epitopes recognized by them were conformational. The characterizations of the selected capture mAbs are shown in Table 1. They were purified, biotinylated and ready for use in the LFI strip test.

**Table 1:** Characterization of capture mAbs against FMDV serotype O, A, and Asia 1

**MAbs**	**Inoculation virus**	**Serotype specificity**	**Isotype**	**Epitope**	**VNT**
F1O-2-5	O1 Campos	O	IgG1k	Conformational	-
F66A_22_-14	A_22_ Iraq	A	IgG2a/k	Conformational	+
F34Asia-8	Asia 1 Shamir	Asia 1	IgG2a/k	Conformational	+
F36Asia-14	Asia 1 Shamir	Asia 1	IgG1/k	Conformational	-

### Immunochromatographic strip test development

**Figure 2:**
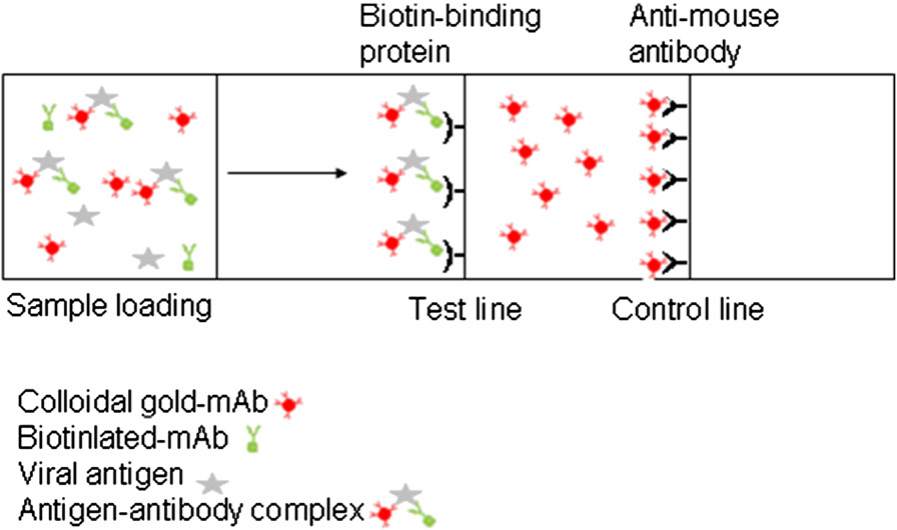
**Schematic diagram of the immunochromatographic strip test using the gRAD.** The test line contains a biotin-binding protein that captures biotinylated capture mAb. The control line contains antibodies that bind the mouse antibody which is responsible for immobilizing the detection conjugate at the control line. When testing, samples were mixed with both the biotinylated capture mAb and the detection colloidal-gold mAb conjugate to form a complex in the running buffer. Then 100 μl of the mixture is applied to the sample well of the gRAD. The mixture migrates along the membrane of the gRAD to the test line which binds and immobilises the biotinylated capture antibody. After 10 minutes, results were determined by visualization. A positive result following the accumulation of complex (biotinylated capture mAb, FMDV and Colloid gold-mAb) is demonstrated by the appearance of reddish-purple bands at both the test line (T) and the control line (C). A negative result is demonstrated by a reddish-purple band at the control line only.

The gRAD was selected and used for the strip test development. In the test, the mixture of the viral antigen with serotype specific biotinlyted capture mAb and colloidal-gold conjugated serotype independent detection mAb were applied to the gRAD and migrated along the membrane. The immune complex reacted with the immobilized biotin-binding protein on the test line making the test line visible, while the remainder reagents continued to migrate. The colloidal-gold conjugated detection mAb bound to the immobilized anti-mouse antibody to form the control line (Figure 2).

Initially, 20 nm colloidal-gold particles were used for conjugation with the detection mAb. However, the sensitivity of the strip test was much lower than that of the DAS ELISA in the preliminary study. Since the diameter of colloidal gold particles may affect the sensitivity of the tests, different sized colloidal-gold particles (20 nm, 40 nm and 60 nm) were examined. With the purpose of sensitivity comparison, a serial dilution of viral antigen was used instead of using protein concentration or TCID50. The test result showed a higher sensitivity with 60 nm colloidal-gold particles conjugated to the detection mAb compared to 20 nm and 40 nm colloidal-gold particles. At a FMDV Asia 1 antigen dilution level of 1:256, only 60 nm colloidal-gold conjugated mAb was positive. The reading results by an ESE-Quant Lateral Flow Reader confirmed the finding by visual assessment (Table 2). Based on the results, 60 nm colloidal-gold particles were selected and utilized in the test. The colloidal-gold conjugated detection mAb at 0.2 μg/test gave the highest sensitivity for viral antigen detection with very low background and without false positive results.

**Table 2:** Comparison of different sizes of colloidal-gold particle conjugated detection mAb in the strip test

**Gold particle size**	**20 nm**		**40 nm**		**60 nm**	
**Antigen dilution**	**ESE reading**	**Visible band**	**ESE reading**	**Visible band**	**ESE reading**	**Visible band**
1:16	109.93	+	244.52	++	226.84	++
1:32	63.07	+	174.40	+	237.03	++
1:64	31.57	+	95.69	+	142.10	++
1:128	16.14	-	43.23	+	88.09	+
1:256	18.79	-	19.21	-	25.43	+

FMDV Asia 1 Shamir (2-fold dilution, 50 μl) was mixed with 1 μl of each biotinylated F34Asia-8, F36Asia-14 and 6 μl mAb (F21-140SO) conjugated with different size of colloidal-gold in the running buffer. The mixture (100 μl) was applied to the gRAD. After 10 minutes, the result was recorded. + +: Strong band; +: Light band; -: No visible band.

### LFI test specificity

**Figure 3:**
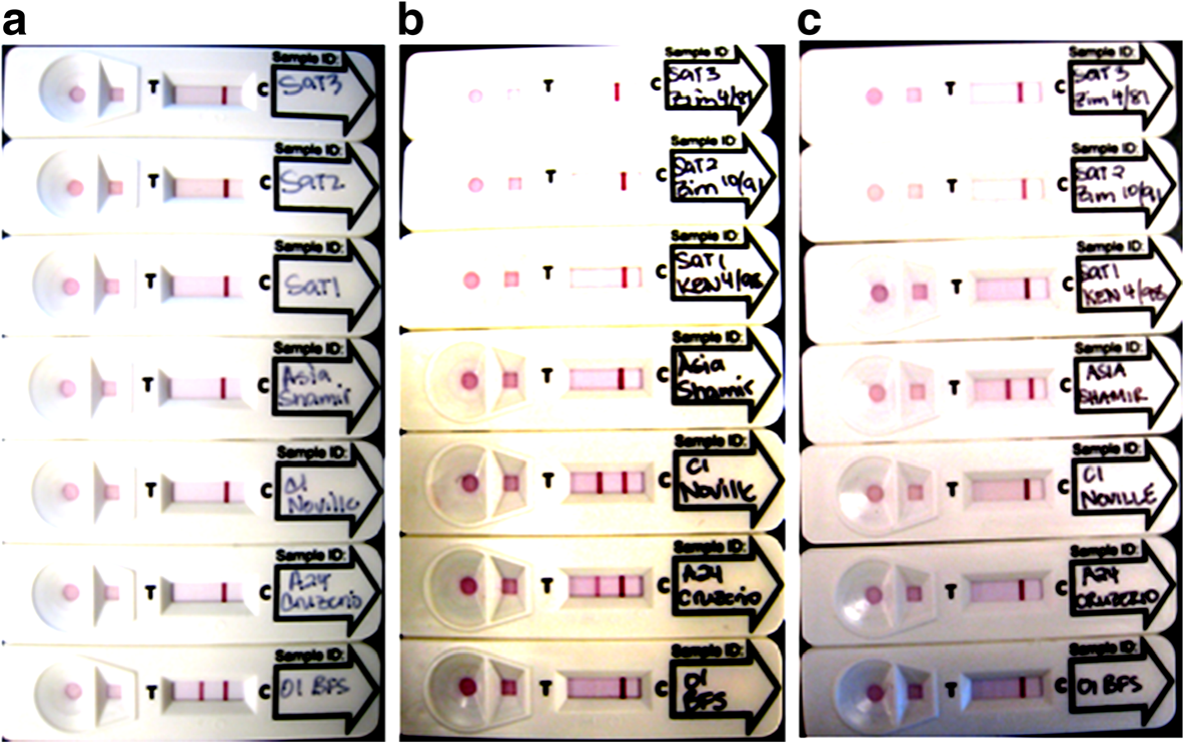
**Specificity of the strip test.** Culture supernatants containing the seven serotypes of FMDV (50 μl) was mixed with 1 μl of biotinylated capture mAbs (F1O-2-5 for serotype O, F66A22-14 for serotype A and F34Asia-8, F36Asia-14 for serotype Asia 1) and 6 μl of colloidal-gold conjugated F21-140SO in the running buffer (50 μl). The mixture (100 μl) was applied to the gRAD. After 10 minutes, the result was recorded. T is the test line and C is the control line. (**a**). The LFI strip test for serotype O; (**b**). for serotype A and (**c**). for serotype Asia 1.

With the intention of determining analytical specificity, culture supernatants of the 7 serotypes of FMDV and of other vesicular disease viruses (Swine vesicular disease viruses; Vesicular stomatitis viruses; Seneca valley viruses; and Vesicular exanthema of swine virus) were examined using the LFI strip tests for each serotype. The specific FMDV viruses demonstrated a strong band on the test line for serotypes O, A and Asia 1 respectively (Figure 3). But the LFI strip test for serotype A demonstrated cross reactivity with 4 out of 9 tested serotype C (C/KEN 1/2004, C/Noville, C/Oberbayern and C/Resende). All other un-related FMDV serotypes and non-FMD vesicular disease viruses showed negative results with no visible band on the test line (Table 3). Thirteen epithelial samples were collected from naïve animals (cattle, pig and sheep). 10% tissue suspensions were prepared and tested using the LFI strip test. All these samples failed to show any visible band on the test line. Because of limited number of negative samples, it is impossible to determine the diagnostic specificity. More negative samples need to be examined.

**Table 3:** The LFI strip test results for FMDV seven serotypes and other vesicular disease viruses

**Virus**	**Serotype O**	**Serotype A**	**Serotype Asia 1**
FMDV O	45/46	0/46	0/46
FMDV A	0/39	39/39	0/39
FMDV Asia 1	0/17	0/17	17/17
FMDV C	0/9	4/9	0/9
FMDV SAT 1	0/11	0/11	0/11
FMDV SAT 2	0/19	0/19	0/19
FMDV SAT 3	0/4	0/4	0/4
SVDV	0/4	0/4	0/4
VSV (IN&NJ)	0/6	0/6	0/6
SVV	0/1	0/1	0/1
VESV	0/1	0/1	0/1

Viruses in culture supernatants (50 μl) were mixed with 1 μl of each biotinylated capture mAb for each serotype and 6 μl of colloidal-gold conjugated F21-140SO in the running buffer. The mixture (100 μl) was applied to the gRAD. After 10 minutes, the result was recorded.

SVDV: Swine vesicular disease viruses; VSV: Vesicular stomatitis viruses; IN: Indiana; NJ: New Jersey; SVV: Seneca valley viruses; VESV: vesicular Exanthema of swine virus.

The ability of the LFI strip test to detect the different isolates for each serotype was examined. All 39 serotype A field isolates were tested demonstrated positive results using the LFI strip test (Table 3). For serotype O, the results showed strong bands on the test line for 44 out of 46 isolates, but a relatively weak band for O/MAY/1/05 and failed to detect O/ECU4/2010 isolate. This virus sample was identified as a positive result using the double polyclonal antibody sandwich ELISA with the optical density (O.D.) reading of 0.231. A mean O.D. value ≥0.1 in the ELISA was measured as positive response. For Asia 1, the test results demonstrated that the strip test was able to identify all the FMDV Asia 1 isolates (n=17) by combining two Asia 1 specific capture mAbs. The LFI strip test has slightly different levels of sensitivity for different Asia 1 isolates. The test line density for six isolates (Asia 1/IRN 31/04; Asia 1/KRG 1/04, Asia 1/VIT 15/05; Asia 1/MYA 1/05 and Asia 1/VIT 8/06) on the strip are lighter than the other tested strains, although virus contents were similar indicated by TCID50/ml. This indicates that the sensitivity of the strip test might be slightly different for diverse FMDV strains. Above results indicated that the LFI strip tests for serotype O and Asia 1 are specific, with no cross reactions with other FMDV serotypes and vesicular disease viruses. While the LFI test for serotype A showed a cross reactivity with 4 isolates of serotype C, but without cross reaction with other vesicular disease viruses.

### LFI test sensitivity

Tissue suspension (10%) made from epithelial lesions collected from pigs experimentally inoculated with the FMDV serotypes O, A and Asia 1 were two-fold serially diluted in PBS. The sensitivity of the strip test was evaluated and compared with that of the DAS ELISA. In the strip test, the densities of the band on the test lines gradually declined from strong to weak indicating that the test is dose dependent (Table 4). ESE reading for the LFI test is correlated with the O.D. value in the DAS ELISA. Samples of 50 μl with a viral titer > 2.55 log10 TCID50/ml for serotype O, > 5.2 log10TCID 50/ml for serotype A and > 6.3 log10TCID 50/ml for Asia 1 produced a visible test line. The LFI strip test for different serotype revealed different test sensitivity. However, the sensitivity of the LFI strip test is equivalent to that of the DAS ELISA for the serotypes O and Asia 1. The LFI strip test for serotype A is one to two dilutions less sensitive than the DAS ELISA.

**Table 4:** Comparison of strip test with DAS ELISA for tissues collected from experimentally inoculated pigs with FMDV

**Pig#/tissue**	**Sample dilution**	**Virus titer log10 TCID50**	**Strip test**		**DAS ELISA**	
			**ESE reading**	**Visual**	**OD**	**Results**
Serotype O						
Epithelial lesion	1:4	4.05	479	++	2.208	+
(Pig14)	1:8	3.75	345	++	1.796	+
	1:16	3.45	252	++	1.207	+
	1:32	3.15	132	++	0.658	+
	1:64	2.85	60	+	0.227	+
	1:128	2.55	27	+	0.168	+
	1:256	2.25	14	-	0.06	-
Serotype A						
Pig 80 Interdigital	1:2	6.7	672	++	2.405	+
	1:4	6.4	534	+	2.298	+
	1:8	6.1	407	++	2.007	+
	1:16	5.8	204	+	1.481	+
	1:32	5.5	83	+	0.884	+
	1:64	5.2	33	±	0.461	+
	1:128	4.9	33	-	0.153	+
Serotype Asia 1						
Coronary	1:2	7.8	NA	++	1.989	+
Band	1:4	7.5	NA	++	1.65	+
	1:8	7.2	NA	++	1.196	+
	1:16	6.9	NA	+	0.746	+
	1:32	6.6	NA	+	0.379	+
	1:64	6.3	NA	+	0.177	+
	1:128	6.0	NA	-	0.055	-

### Validation of the LFI strip test with samples from experimentally inoculated animals

To evaluate whether the LFI strip tests for three serotypes are able to detect FMD viral antigens in clinical samples, tissue suspensions and vesicular fluid collected from experimentally inoculated animals with FMDV serotypes O, A and Asia 1 were tested using the LFI strip test, DAS ELISA and RRT-PCR [18]. As shown in Table 5, all samples of vesicular fluids and epithelia suspensions (tongues, interdigital areas and coronary bands) collected from these animals were identified as positive by the LFI strip test, DAS ELISA and RRT-PCR. The use of 1 μl of vesicular fluid in the LFI strip test also demonstrated a positive result.

**Table 5:** FMDV serotype O, A, and Asia 1 detection using different methods for samples collected from experimentally inoculated animals

	**Strip test**	**ELISA**	**RRT PCR**
	Pos/total*	Pos/total*	Pos/total*
Serotype O			
Pig			
Tongue	4/4	4/4	4/4
Epithelial lesion	4/4	4/4	4/4
Interdigital area/coronary band	2/2	2/2	2/2
Vesicular fluid	2/2	2/2	2/2
Cow			
Tongue	2/2	2/2	2/2
Coronary band	1/1	1/1	1/1
Interdigital area	2/2	2/2	2/2
Vesicular fluid	2/2	2/2	2/2
Serotype A (pig)			
Tongue	4/4	4/4	4/4
Coronary band	4/4	4/4	4/4
Interdigital area	4/4	4/4	4/4
Vesicular fluid	1/1	1/1	1/1
Serotype Asia 1 (pig)			
Tongue	2/2	2/2	2/2
Coronary band	4/4	4/4	4/4
Interdigital area	4/4	4/4	4/4
Vesicular fluid	4/4	4/4	4/4

Pos/total: Tested positive numbers/total tested samples.

**Figure 4:**
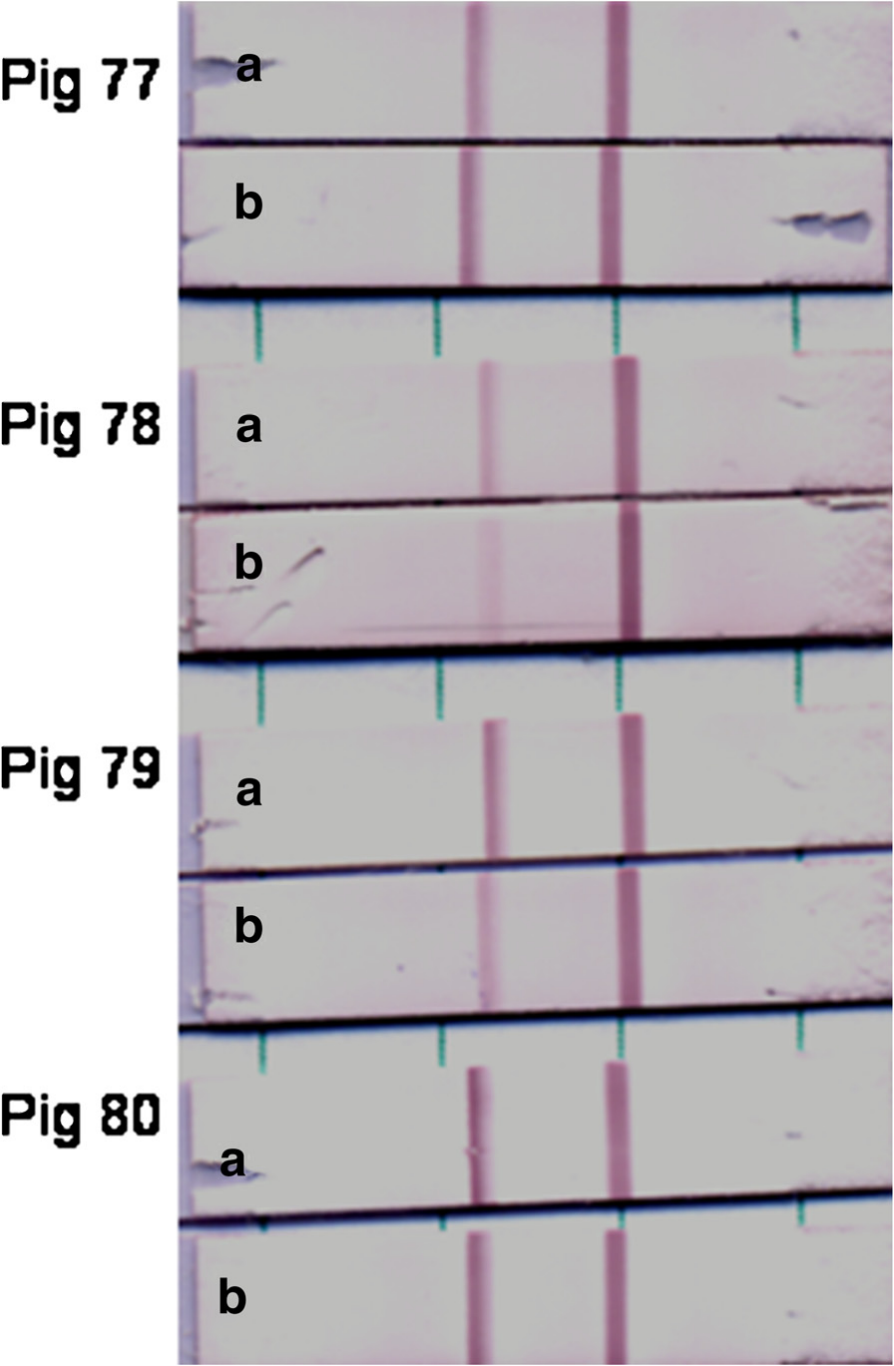
**Comparison of LFI test for tissue suspensions and lesion swabs.** Tissue suspensions and flocked swabs over lesions collected from 4 experimentally inoculated pigs with A_22_ Iraq. Samples (**a**. tissue suspension, **b**. lesion swab sample, 50 μl) were mixed with 1 μl of biotinylated F66 A_22_-14 and 6 μl of colloidal gold conjugated F21-140SO in the running buffer. The mixture (100 μl) was applied to the gRAD. After 10 minutes, the result was recorded.

The aim of the current project is to apply the LFI strip test in the field. However, preparation of 10% tissue suspensions on site may be difficult. To make sample preparation procedure straightforward, samples collected from or over the ruptured lesion areas using nylon flocked swabs were tested. A total of 11 swab samples were collected from 4 pigs experimentally inoculated with FMDV A_22_ /Iraq. All of them demonstrated positive results using the LFI serotype A strip test and the DAS ELISA (Table 6). The test results of swab samples were also compared with tissue suspension (Figure 4). It was observed that viral antigens in both tissue suspension and swab samples obtained around the lesion area demonstrated positive bands on the test lines, and two of swab samples showed lighter testing bands than 10% tissue suspensions. It is demonstrated that this procedure is simple and can be easily accomplished in the field. To conclude, the strip tests for FMDV serotypes O, A, and Asia 1 are specific and suitable for detecting the FMDV antigen in vesicular fluid, epithelia suspensions and swabs collected over the lesion area.

**Table 6:** **LFI strip test and DAS ELISA results of swab samples collected from pigs experimentally inoculated with FMDV A**_**22 **_**/Iraq**

		**LFI strip test**	**DAS ELISA (OD)**
Pig 77	Swap 1	+	1.717
	Swap 2	+	1.3
	Swap 3	+	0.307
Pig 78	Swap 1	+	0.604
Pig 79	Swap 1	+	1.589
	Swap 2	+	0.833
	Swap 3	+	2.04
Pig 80	Swap 1	+	3.079
	Swap 2	+	3.372
	Swap 3	+	2.713
	Swap 4	+	2.918

### Amino acid alterations on mAb binding site of the virus cause the failure of viral detection

**Figure 5:**
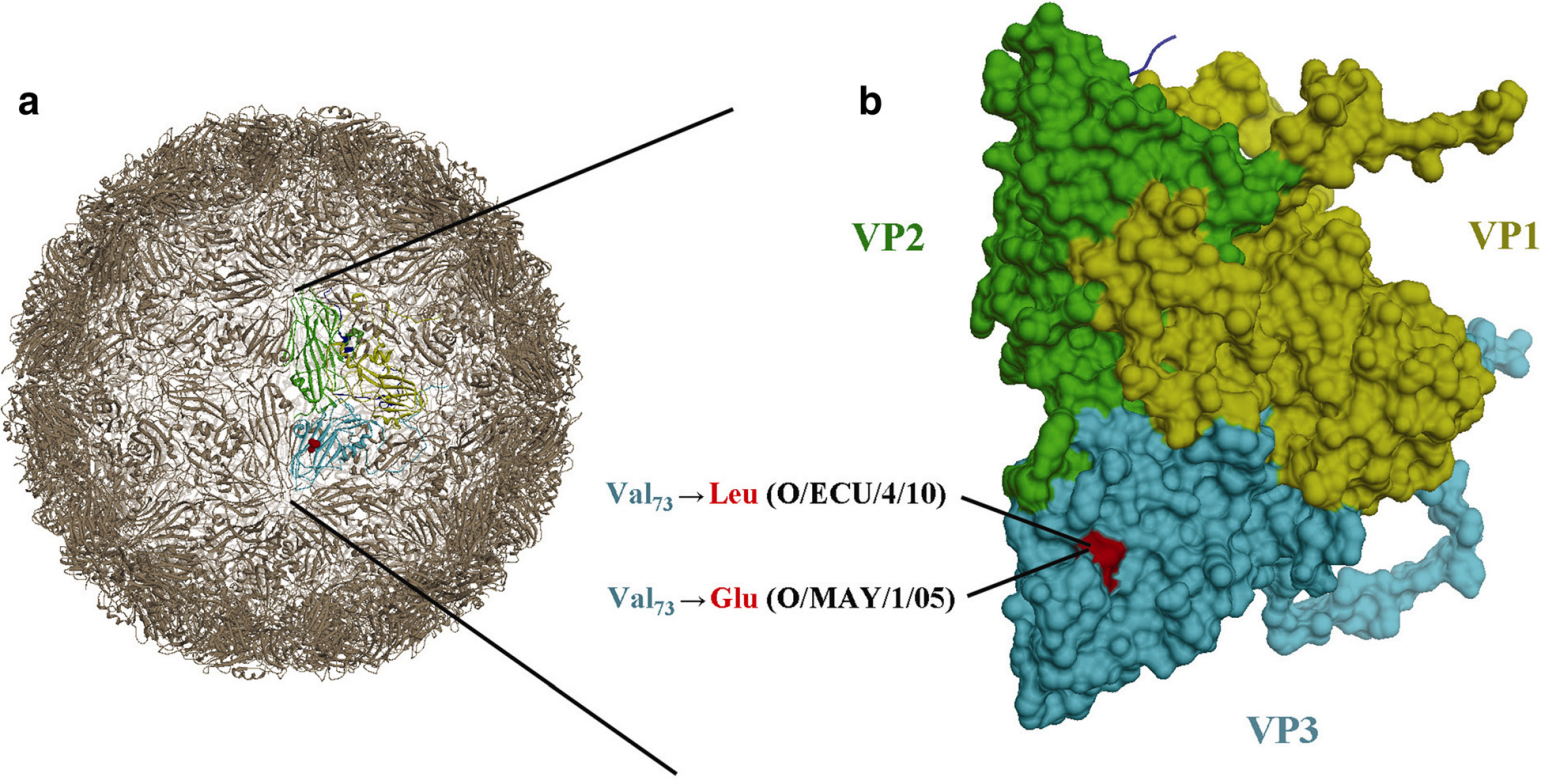
**Localization of a unique amino acid change at same site shared by FMD O/ECU/4/10 and O/MAY/1/05 in capsid protein.** (**a**) Low-resolution overview of the reduced serotype O capsid structure (prepared with Chimera). (**b**) Blow-up of capsid crystal asymmetric unit (PDB # 1FOD) corresponding to colored region in “A” consisting of one VP1 protein (yellow), one VP2 protein (green), and one VP3 protein (cyan) depicted as surface model and one VP4 protein (blue) in cartoon format, with O/ECU/4/10 (Val_73_→Leu) and O/MAY/1/05 (Val_73_→Glu) unique mutations shown in red.

The mAb F1O-2-5 used in the LFI strip test for serotype O was unable to react with isolate O/ECU/ 4/10 and showed weak binding with O/MAY/1/05. Therefore, the P1 gene sequences of the two isolates were sequenced and compared with those of the FMDV O1/Campos and 43 additional FMDV O viruses (unpublished data). Six unique mutations in VP1, 4 of them located on previously identified antigenic sites [19]; three unique mutations in VP2, and seven unique mutations in VP3 (1 on antigenic site) were observed for O/ECU/4/10 [GenBank: KC519630] One unique mutation in VP3 at amino acid position 73 was found to be shared by O/ECU/4/10 (Val→Leu) and O/MAY/1/05 (Val→Glu) [GeneBank: KC519631] (Figure 5). Amino acid alteration at this position may led to loss of binding capacity of mAb with O/ECU/4/10 and reduced binding capacity of mAb with O/MAY/1/05 in the LFI strip test. This indicated that the binding epitope of the capture mAb for serotype O F1O-2-5 is located in VP3 around amino acid position 73.

## Discussion

Outbreaks of FMD have resulted in tremendous economic losses. Thus, the development of a rapid and easily performed test for FMD detection is important for controlling a FMD outbreak and containing its spread. The LFI strip test was developed as a method suited for laboratories and on-site for rapid FMD viral antigen detection. Monoclonal antibodies were used for this test because the use of mAbs in the test increases the specificity, accuracy, consistency and efficiency of diagnosis compared with polyclonal antibodies. Monoclonal Abs were produced from mice inoculated with BEI-inactivated FMDV serotypes O, A, or Asia 1. Based on the DAS ELISA results, mAbs F1O-2-5, F66A_22_-14 and F34Asia-8, F36Asia-14 were selected and used as the capture mAbs for serotype O, A, Asia 1 LFI strip tests respectively.

The selected serotype A capture mAb reacted with all tested field isolates, while the serotype O capture mAb failed to react with one (O/ECU4/10) out of 46 isolates. The two Asia 1 specific mAbs demonstrated low binding affinity to some strains and failed to react with at least one of the tested strains. To increase the sensitivity and detection range of Asia 1 strains, the combination of the two FMDV Asia 1 specific mAbs were used as the capture antibody. This would overcome the limitation of a single mAb binding one epitope since using only one capture mAb may reduce the ability to recognize viruses when antigenic evolution occurs. The capture mAbs selected for O and Asia 1 were shown to react with FMDV specific serotype, while the mAb used for serotype A demonstrated cross-reactivity with four of the nine tested serotype C isolates. The mAb F66A_22_-14 was selected for serotype A since it is the only mAb that reacts with 39 field isolates. Although this mAb showed cross-reactivity to some of the serotype C isolates, it is better than missing isolates when used for diagnosis. In addition, no FMD serotype C outbreak has been reported since 2005.

A FMDV serotype independent mAb F21140SO-42 [17] was used as the detector in the strip test. Using one serotype independent mAb as detection antibody has an advantage for the future development of a multi serotype LFI strip test using a single device. An important consideration in designing an LFI strip test when using mAbs is that the capture and detection antibodies should recognize different non-overlapping epitopes. When the antigen binds to the capture antibody, the epitope recognized by the detection antibody should not be hidden. DAS ELISAs were used to confirm that the antibody pair (capture and detection) did not interfere or compete with the same binding epitope before the LFI test development. This will prevent reduced sensitivity in the test.

The detection mAb was conjugated to colloidal gold particles using Naked Gold in a Box™. The kit is easy to use for preparation of highly reactive gold conjugates. The procedure allows us to quickly determine the optimal colloidal gold binding conditions for attaching to the Fc portion of mAbs. The resulting gold particles are highly active and can be directly used in strip tests. The test result showed a higher sensitivity using 60 nm colloidal gold particles conjugated with the detection mAb compared with 20 and 40 nm colloidal gold particles. At an antigen dilution level of 1:256, only 60nm gold particles scored positive results. 60nm colloidal gold particles were selected and used for the study. This is a clear indication that the density of color showing on the strip is closely related to the size of the colloidal gold particles.

Analysis of the specificity demonstrated that the strip tests for serotype O and Asia 1 were specific. The samples containing the other serotypes of FMDV as well as other vesicular disease viruses were all negative in the strip test (Table 3). The LFI strip test for serotype A showed positive results for four serotype C field isolates, besides the serotype A samples. The reason for the cross reactivity is uncertain. It is possible that some mutations occurred close to the mAb binding epitope in these serotype C isolates. Thirteen negative epithelial samples were tested using the LFI strip test for all three serotypes in the current study. It is not possible to determine the diagnostic specificity, because of small number of negative samples tested. The sensitivity of the LFI strip test is equivalent to that of the DAS ELISA for the serotypes O and Asia 1 using the tissue samples collected from experimentally inoculated pigs. The LFI strip test for serotype A is one to two dilutions less sensitive than the DAS ELISA. The LFI strip test’s sensitivity is similar to the results reported by Lin et al. [20].

The aim for the LFI strip test is to detect FMDV viral antigens from clinical samples. Vesicular liquids and epithelia collected from the experimentally inoculated animals (pigs and cows) were examined and the LFI strip test correctly revealed positive results. To obtain full validation of the strip test more of these samples need to be tested since no field samples were examined in the current study. Similar to the ELISA, the sensitivity of the LFI strip test is lower when compared with the RRT-PCR [21]. Both the LFI strip test and the DAS ELISA were unable to consistently detect viral antigen in the samples collected from nasal swabs and sera which were positively identified by the RRT-PCR. This result is consistent with Ferris’s finding that the LFI strip test is suitable for the detection of FMDV in vesicular fluid or epithelia due to limitation of test sensitivity [13]. Therefore, it is not suitable to use the LFI test for virus detection in nasal swabs and serum samples where the number of virus particles may not be sufficient for both the LFI strip test and ELISA.

Ferris [13] indicated that for epithelia, the sample must be homogenized to release virus antigen from the tissue. However it is difficult to prepare 10% tissue suspensions in the field. To make the sample preparation procedure straightforward, swab samples collected over the lesion area were examined. All of them (n= 11) demonstrated positive results using the serotype A LFI strip test and the DAS ELISA. It is possible that viruses were released because vesicles often rupture rapidly and becoming erosions. This makes the sample preparation procedure simpler than preparing the 10% epithelia suspension and can be easily done in the field. The use of flocked swabs increased surface area which might result in better virus recovery rates than when using the conventional swabs [22]. A combination of two swabs per vial for this study may also increase the amount of viral particles, thus enhancing test sensitivity.

The strip test has many advantages including low cost, short timeline for development of results, ease of result interpretation and no requirement for special equipment or highly trained personnel. However, it also has limitations that include the inability to provide qualitative or semi-qualitative results. In this study, an ESE lateral flow reader was used to confirm the results by visual reading. A good correlation between the readout by the reader and visual reading was observed. However, some false readings were observed using the ESE reader. Some negative samples without a visible band on the test line showed a reading higher than some of the positive samples with visible bands. A possible explanation for the false positive may be position errors in the reading window and device housing making it difficult to determine the cut-off value. For research purposes, the ESE reading value can be used to confirm visual reading and calculate the correlation with other test results.

The capture mAb used for serotype O in the LFI strip test does not react with O/ECU/4/10 and has a low binding affinity with O/MAY/1/05. To provide a completed picture at nucleotide level for capsid genes, the P1 regions of the two isolates that encode capsid proteins were sequenced and compared with those of mAb derivative strain O1/Campose and additional 44 field isolates available in the laboratory. In consistence with VP1 sequences reported by Maradei [28], O/ECU/4/10 had an amino acid deletion near the antigenic site 1 within the VP1 GH loop. In their report, the mAb profiling showed loss of reactivity of most of the O/ECU 2010’s viral samples with four mAbs, three of them with neutralizing properties. Levels of protective antibodies induced by the vaccine against the field strains also pointed to a loss of a protective response against O/ECU4/10. The sequence data revealed that O/ECU/4/10 has many unique amino acid alterations within the surface capsid proteins (i.e. VP1, VP2, and VP3). Interestingly however, the amino acid change in VP3 at position 73 was only observed from O/ECU/4/10 and O/MAY/1/05. It is assumed that the epitope of mAb F1O-2-5 applied in the strip test resides on amino acid position around 73 in the VP3 protein. The changes in the binding site because of the amino acid substation led to loss of binding capacity of O/ECU/4/10 and reduced binding affinity of O/MAY/1/05 with mAb F1O-2-5. More mAbs against FMDV serotype O will be produced and screened. Eventually, one mAb recognizing a conserved epitope, thus reacting with all field isolates will be produced and used in the LFI test.

All of the mAbs used in the LFI strip test recognize conformational epitopes since they do not bind denatured viruses in the indirect ELISA. If the virus in the sample is denatured during environmental conditions or transportation, the denatured viruses will not be detected using this LFI strip test. The sensitivity of the strip test may be slightly different for diverse FMDV strains. An explanation for this may be that FMD viruses undergo frequent mutations that result in changes of structural proteins. Conformations of binding epitopes may also change in the process. The binding affinity of mAbs would be reduced against changed epitopes as all mAbs used in the LFI strip test recognize conformational epitopes.

## Conclusions

The LFI strip tests for FMDV serotypes O, A and Asia 1 have been developed and achieved sensitivity similar to that of the ELISA in a procedure that takes only 10 minutes. The serotype specific capture mAbs (O, A and Asia 1) and one serotype independent detection mAb with high binding affinity to all FMDV serotypes are crucial to the high specificity and sensitivity requirement for the strip test development. The gRAD and the colloidal conjugated detection mAb are suited for different serotype strip tests without modification of the device. The LFI strip tests developed in this study have great potential for on-site FMDV serotypes O, A, and Asia 1 diagnosis to confirm clinical findings in FMD-endemic areas lacking highly specialized staff or can be used as a screen test in laboratories. However, the newly developed LFI strip test needs to be further standardized to achieve optimal diagnostic sensitivity and specificity. The current LFI strip test allows the detection of a single serotype using each gRAD device. A FMDV multiple serotype antigen detection using a single device will be developed where the capture mAbs will be labelled with different tags and the serotype independent detection mAb will be used. The ability of the LFI strip test to produce rapid results and high specificity makes it a valuable tool for early diagnosis of FMD.

## Materials and methods

### Ethics statement

The animal use and procedures in this study was approved by the Canadian Science Centre for Human and Animal Health Animal Care Committee (Animal use document #C-11-003).

### Preparation of FMDV

All of the FMDV isolates used in this study were obtained from the FAO/OIE World Reference Laboratory for FMD at the Pirbright Institute, Pirbright, UK.

Baby hamster kidney clone, 21 cells (BHK-21) were cultured in Glasgow’s MEM supplemented with 2 mM L-glutamine and 50 μg/ml gentamycin and infected with FMDV. Viruses were harvested 24 hours post-infection and clarified by centrifugation at 8000 g for 30 minutes.

For inactivation of the virus, 10 mM 2-Bromoethylamine Hydrobromide (BEI) was added to cell culture supernatants containing viruses and inactivated for 24 hour at 37°C. After 24 hours, any remaining BEI was inactivated using Sodium Thiosulphate to a final concentration of 2%. The inactivated FMDV Asia 1 Shamir strain was concentrated and purified for mice inoculation. Briefly, tissue culture supernatant containing virus was mixed with 50% polyethylene glycol (PEG, MW 8000) to a final concentration of 7.5% and stirred for 12 hours at 4°C. The precipitate was obtained by centrifugation and resuspended in Tris-NaCl Buffer (150 mM NaCl, 50 mM Tris pH 7.8). The concentrated virus was layered onto 15-45% sucrose density gradient and ultracentrifuged at 27,000 rpm for 2.5 hours at 4°C. The whole virus (140S) band was collected.

Denatured virus was prepared by adding 10 μl of 10% SDS and 10 μl of 1M Dithiothreitol (DTT) to 100 μl of purified virus. The mixture was heated for 5 minutes at 95°C. The denatured antigen was then diluted to assay concentrations in a buffer and used for coating antigens in indirect ELISAs with no further treatment.

### Production of monoclonal antibodies

Mice immunizations and mAb production were performed as previously described [17]. Briefly, female BALB/C mice were inoculated subcutaneously with purified and BEI inactivated FMDV (20 μg/mouse) in an equal volume of TiterMax Gold (TiterMax USA Inc., Norcross, USA). Two to three identical boosts were administered at four week intervals. Mice were boosted with the same antigen in phosphate-buffered saline (PBS) by intravenous injection 3–4 days prior to the mice being euthanized for the purpose of spleen cell collection. Immunized spleen cells were fused with myeloma cells (P3X63 Ag8.653, ATCC, Rockville, MD, USA). After 2 weeks, hybridoma supernatants were screened using FMDV serotype specific DAS ELISAs. The positive clones were subcloned using a limiting dilution method. Isotyping was performed, using a mouse monoclonal antibody isotyping kit (Roche, Indianapolis, IN, USA).

### Purification of monoclonal antibody

The hybridomas were grown in BD Cell MAb medium (Becton, Dickinson and company, MD, USA) supplemented with 2% fetal bovine serum. After 7 days culture, the culture supernatants were harvested and concentrated. The mAbs were purified from hybridoma culture supernatants by a HiTrap Protein-G affinity column (GE, Fairfield, CT, USA) using an AKIA chromatography system according to manufacturer’s instruction.

### Biotinylation of mAb

The purified mAb was dialyzed against 0.1 M NaHCO_3_, pH 8.4 at 4°C. A biotin-spacer conjugate (D-Biotinoyl-ϵ-Aminocaproic Acid N-hydroxysuccinimide Ester, Sigma-Aldrich, St Lucia, MO, USA) was dissolved in anhydrous DMSO (Sigma-Aldrich, St Lucia, MO, USA) at 8 mg/ml and then added to the antibody (Biotin-spacer:mAb, 80 μg:mg). The reaction was allowed to proceed for 1 hour at room temperature in the dark, following which unbound biotin was removed by extensive dialysis against PBS at 4°C. The reagent was stored at 4°C in PBS with 0.01%NaN_3_.

### Double antibody sandwich (DAS) ELISA for detection antibody selection

Microtitre plates (Nunc-Immunoplate Maxisorp, Roskilde, Denmark) were coated with purified anti-FMDV Asia 1 mAbs diluted in 0.06 M carbonate/bicarbonate buffer, pH 9.6 and incubated overnight at 4°C. The plates were blocked with 5% skim milk in PBS with 0.05% Tween 20 at 37°C for 1 hour. The FMDV Asia 1 antigen diluted in PBS with 5% skim milk and 0.05% Tween 20 was added to the plates and then the biotin-conjugated FMDV serotype independent mAbs were added. After an incubation step, the horseradish peroxidase (HRP)-conjugated strept-avidin (1:2000, Jackson ImmunoResearch Laboratories, West Grove, PA, USA) was used and then O-phenylenediamine dihydrochloride (OPD, Sigma-Aldrich, St Lucia, MO, USA) was added. An equal volume of 2.0 M sulfuric acid was added to each well to stop the color reaction. The optical absorbance was measured at 490 nm using an automated plate reader (Photometer Multiskan Reader, Labsystems, Foster, VA, USA). Each incubation step was 60 minutes at 37°C with gentle shaking and followed by washing three times with washing buffer.

### Colloidal-gold conjugate detection mAb

The detection mAb was conjugated to colloidal gold particles using a Naked Gold in a Box™ (Rapid Assay, Copenhagen, Denmark). For optimal binding of the antibody, the pH of the gold solution was adjusted to 7.8, slightly above the iso-electric point of the mAb through a series of pH titration with buffers. The mAb (33 μg in PBS) was mixed with 1 ml of colloidal-gold solution. The mixture was stirred vigorously for 30 minutes and then 100 μl of the blocking buffer was added, followed by incubation at room temperature overnight. The resulting conjugated colloidal-gold mAb is ready for use.

### Animal experiments

All procedures involving experimental animal inoculations and care were performed following the Canadian Council of Animal Care guidelines. Four pigs for each serotype (O, A and Asia 1) and two cows for serotype O were inoculated with BHK-21 cell culture supernatant containing FMDV O/UKG/34/2001, A/IRN/1/2009 or Asia 1/PAK20/2003 (6–7 log10 50% tissue culture infecting dose (TCID50/ml) /animal) through the intra-lingual (cattle), the heel bulb (pigs) and the coronary band. Vesicular fluid, coronary band, interdigital epithelium and tongue were collected on 3–4 days post inoculation (dpi) in 5 ml phosphate buffered transport medium.

### Tissue sample preparation

The tissue samples (1g) for testing were emulsified using a sterile mortar and pestle with Dulbecco's PBS as diluent to make a 10% Weight/Volume suspension [23]. The suspension was clarified by centrifugation at 2000 x g (3000 rpm) at 4°C for 20 minutes and the supernatant was harvested. An antibiotic stock solution was added to the supernatant to obtain 10% concentration. The mixture was incubated at room temperature for 30 minutes. This preparation is the undiluted 10^0^ sample.

Nylon flocked swabs (Puritan medical products company LLC, USA) were used for collecting swab samples over ruptured lesion areas. The dried nylon flocked swabs were rubbed and rolled firmly several times across or over the lesion area. Two swabs from each lesion area were inserted into a tube containing 1 ml PBS. The samples were stored at −70°C until testing.

### Immunochromatographic strip test

The RapidAssay Devices were purchased from the Rapid Assays (Copenhagen, Denmark). The test line contains a biotin-binding protein that captures both complexed and free biotinylated capture mAb. The control line contains antibodies that bind the mouse antibody which is responsible for immobilizing the detection conjugate at the control line. When testing, virus in samples were mixed with the serotype specific biotinylated capture mAb and the detection colloidal-gold mAb conjugate to form a complex in the running buffer (Rapid Assays, Copenhagen, Denmark). Then 100μl of the mixture was applied to the well of the gRAD. The mixture migrated along the membrane of the gRAD to the test line which binds and immobilises the biotinylated capture antibody. After 10 minutes, results were determined either by visualization or an ESE-Quant Lateral Flow Reader (ESE GmbH, Stockach, Germany). A positive result following the accumulation of complex (biotinylated capture mAb, FMDV antigen and Colloid gold-mAb) was demonstrated by the appearance of reddish-purple bands at both the test line (T) and the control line (C) (Figure 2). A negative result was demonstrated by a reddish-purple band at the control line only.

### Sequencing FMDV O P1 gene

Genomic RNA was extracted from laboratory stocks using the Rneasy Mini Kit (Qiagen, Venlo, Netherlands). Terminal oligonucleotide primers complementary to the L gene (5’-TTCTGGTGTTTGTCCCGTACGAT-3’) (Invitrogen, Carlsband, USA) and 2B gene (5’-GTTGACATGTCCTCCTGCATCTG-3’) for reverse transcription-PCR and additional ones for internal sequencing were chosen from the most conserved sequence region by alignments of available FMDV O whole genome sequences from GeneBank. Full-length cDNA copies of the P1 genes of each virus were synthesized from genomic RNA by using the terminal primers and SuperScript™ III reverse transcriptase (Invitrogen, Carlsband, USA). The cDNAs were amplified by PCR using the Expand™ Long Template PCR system according to the manufacturer’s instruction (Roche, Indianapolis, USA). PCR products used for DNA sequencing were gel purified using QIAquick® gel extraction kit according to the manufacturer’s instruction (Qiagen, Venlo, Netherlands). DNA sequencing was performed in both directions by use of an ABI Prism BigDye Terminator v3.1 Cycle Sequencing Ready Reaction kit (Applied Biosystems, Carlsbad, California, USA) and an Applied Biosystems Genetic Analyzer DNA Model 3130X. Sequences obtained from both directions were assembled and checked for accuracy with SeqMan® (Lasergene®, Version 9; DNASTAR, Inc.). Pairwise nucleotide sequence alignments were performed using the Martinez-NW method [24] and the Lipman-Pearson method [25] for protein alignments in MegAlign® (Lasergene).

### 3D structural analyses

Molecular graphics coordinates of the FMDV O1/BFS1860 crystal structure (PDB # 1FOD) [26] were manipulated with the UCSF Chimera package from the Resource for Biocomputing, Visualization, and Informatics at the University of California, San Francisco [27]. The resulting images were imported into Adobe Photoshop for editing.

## Competing interests

The authors declare that they have no competing interests.

## Authors’ contributions

MY: designed and supervised all experiments, produced monoclonal antibodies, developed the lateral flow strip test, tested the virus isolates for FMDV serotype A and drafted the manuscript. MG: performed the ELISAs for mAb selection, tested the virus isolates for serotype Asia 1 and O. WX: performed sequencing and the sequencing alignments, located mAb biding sites on the FMDV crystal structure. HB: assisted mAb production. AC: provided the FMD antigen for mAb production and participated in mAb production. All authors have read and approved the final version of the manuscript.
